# Pathogenicity of *Shigella* in Chickens

**DOI:** 10.1371/journal.pone.0100264

**Published:** 2014-06-20

**Authors:** Run Shi, Xia Yang, Lu Chen, Hong-tao Chang, Hong-ying Liu, Jun Zhao, Xin-wei Wang, Chuan-qing Wang

**Affiliations:** Collage of Animal Science and Veterinary Medicine, Henan Agricultural University, Zhengzhou, People's Republic of China; University of California Merced, United States of America

## Abstract

Shigellosis in chickens was first reported in 2004. This study aimed to determine the pathogenicity of *Shigella* in chickens and the possibility of cross-infection between humans and chickens. The pathogenicity of *Shigella* in chickens was examined via infection of three-day-old SPF chickens with *Shigella* strain ZD02 isolated from a human patient. The virulence and invasiveness were examined by infection of the chicken intestines and primary chicken intestinal epithelial cells. The results showed *Shigella* can cause death via intraperitoneal injection in SPF chickens, but only induce depression via crop injection. Immunohistochemistry and transmission electron microscopy revealed the *Shigella* can invade the intestinal epithelia. Immunohistochemistry of the primary chicken intestinal epithelial cells infected with *Shigella* showed the bacteria were internalized into the epithelial cells. Electron microscopy also confirmed that *Shigella* invaded primary chicken intestinal epithelia and was encapsulated by phagosome-like membranes. Our data demonstrate that *Shigella* can invade primary chicken intestinal epithelial cells in vitro and chicken intestinal mucosa in vivo, resulting in pathogenicity and even death. The findings suggest *Shigella* isolated from human or chicken share similar pathogenicity as well as the possibility of human-poultry cross-infection, which is of public health significance.

## Introduction

*Shigella* is a genus of gram-negative, facultative anaerobic Enterobacteriaceae that includes *S. dysenteriae*, *S. flexneri*, *S. boydii*, and *S. sonnei*. *Shigella* species have highly evolved invasive systems that enable the bacteria to invade and multiply within the human intestinal epithelia, ultimately leading to severe inflammatory colitis called bacillary dysentery or shigellosis. Shigellosis remains a worldwide health concern. Statistics from 1966–1997 estimated that over 1.1 million deaths were caused by shigellosis each year worldwide, the vast majority (0.88 million) of which occurred in Asia [1]. Another report based on 1990–2009 statistics estimated that 125 million people in Asia were infected with *Shigella*, resulting in 14,000 deaths annually, most of which were children under 5 years of age [2].

The natural hosts of *Shigella* are conventionally humans and other primates. However, reports of *Shigella* infection in new hosts including monkeys, rabbits, calves, and piglets have emerged [3]–[6]. In 2004, our team first reported chicken shigellosis, characterized by bloody and purulent dysentery in chickens [7]. A serum epidemiological survey of poultry flocks in China with a history of dysentery showed that the seroprevalence of *Shigella* was 28.3–33.7%, suggesting that *Shigella* is an important etiology of chicken dysentery in China [8]. *Shigella* species isolated from human or chicken have identical biological and serological characteristics [9] and are highly genetically homogeneous, suggesting that *Shigella* isolated from chicken may be a descendant or a new subtype of *Shigella* isolated from human [10].

To determine the origin of *Shigella* isolated from chicken and its pathogenicity in humans and therefore identify potential cross-infection between humans and poultry, it is necessary to infect primates with *Shigella* isolated from chicken and study its pathogenicity. However, such animal models have not been established and would be extremely difficult to perform. In this study, specific pathogen-free (SPF) chickens and primary chicken intestinal epithelial cells were infected with *Shigella* isolated from human to investigate the pathogenicity and invasive capacity of *Shigella* in chickens, the origin of *Shigella* isolated from chicken, and the possibility of *Shigella* cross-infection between humans and poultry.

## Materials and Methods

### Ethics statement

This study was carried out in strict accordance with the Guidance Suggestions for the Care and Use of Laboratory Animals formulated by The Ministry of Science and Technology of the People's Republic of China. The protocol was approved by the Institutional Animal Care and Use Committee at The University of Henan Agriculture. All surgery was performed under sodium pentobarbital anesthesia, and all efforts were made to minimize suffering.

### Culture of the *Shigella* strain ZD02

The *Shigella* strain ZD02 (identified as *S. flexneri* serotype 2a) [11] was isolated from stool from a male donor whose clinical symptoms were diarrhea and bloody dysentery following informed consent. Written consent was obtained from subjects in accordance with approved ethics committee of medical research, First Affiliated Hospital of Zhengzhou University and HIPPA regulations. These bacteria were inoculated in Luria-Bertani (LB) broth and grown with shaking overnight at 37°C. Then the bacteria were subcultured into fresh LB broth at a volume ratio of 1∶30 and cultured with shaking at 200 rpm for 3 h at 37°C. The cells were pelleted by centrifugation at 12,000×*g* for 5 min at 4°C and washed three times with phosphate-buffered saline (PBS, pH 7.4). The pellet was then resuspended in antibiotic-free culture medium.

### Virulence determination of the *Shigella* strain ZD02

To determine its virulence and invasiveness on epithelial cell, the strain ZD02 was subjected to a keratoconjunctivity assay (Sereny test) and a HeLa cell invasiveness ability test. The Sereny test using guinea pigs was performed as described previously [12]. Female Hartley guinea pigs ranging in weight from 350 to 400 g were purchased from Zhengzhou University. Two guinea pigs were randomized for inoculation with ZD02, and one inoculated with LB broth as a control. Briefly, a 20-µL bacterial suspension at a density of 1.6×10^10^ CFU/mL was inoculated into the conjunctival sacs of guinea pigs anesthetized with an intraperitoneal injection of sodium pentobarbital (0.6 mg/10 gr). The areas surrounding the eyes were gently rubbed to distribute the medium evenly. Pathological changes in the guinea pig eyes were observed at 4 h, 24 h, 48 h, and 72 h. Abiding to the most stringent animal welfare standards [13], human endpoints were applied. The guinea pigs were euthanized using T61 intravenously at 72 h after the inoculation with the occurrence of signs of appetite loss, lethargy, and listlessness.

ZD02 cells were suspended in Dulbecco's Modified Eagle's Medium supplemented with Ham's F12 (DMEM/F12; Gibco, USA) and inoculated into HeLa cells at a ratio of 10∶1 [14]. Two hours later, the HeLa cells were digested with trypsin and centrifuged. The pellet was fixed with 2.5% glutaraldehyde and the bacterial invasive capacity in HeLa cells was observed using transmission electron microscopy (TEM) (JEM-1400; Japan).

### Pathogenicity of the *Shigella* strain ZD02 in three-day-old SPF chickens

Three-day-old SPF male chickens (license SYXK (Yu) 2011–0010, Sipafas Poultry, Jinan, China) were infected with the *Shigella* strain ZD02 via intraperitoneal or crop injection. Each infection route contained five subgroups of 10 chickens each. The bacterial suspension at a density of 6×10^9^ CFU/mL was serially diluted into five concentrations at 10× intervals. Each animal was inoculated with a 0.5-mL bacterial suspension. Another 10 chickens inoculated with 0.5 mL of LB broth via either route as controls (Table S1). The clinical signs were observed every 6 h. After 48 h post-infection, some chickens showed non-transient hypothermia, appetite loss, severe distress and impending death. Abiding to the most stringent animal welfare standards, human endpoints were applied when animals were moribund and severely cachectic. The moribund chickens infected with the *Shigella* ZD02 were euthanized using T61 intravenously (0.3 ml/kg) (Intervet, Ukkel, Belgium). The median lethal dose (LD50) was calculated using the Reed-Muench method. The dead chickens were dissected for observation of the gross pathological changes. All of the surviving chickens infected with the *Shigella* ZD02 were euthanized using T61 intravenously at 120 h after the inoculation and dissected for observation as well.

### Invasiveness of the *Shigella* strain ZD02 in chicken intestinal epithelial cells

Eight one-day-old SPF chickens were randomized for inoculation with ZD02. The animals were anesthetized with an intraperitoneal injection of sodium pentobarbital (0.6 mg/10 gr). Anesthesia was maintained with 1% isoflurane in conjunction with 1% pure oxygen using a semi-closed circuit. Intramuscular injection of fentanyl was used for pain management. Midline incisions were made in the chickens and the intestinal loops were created by ligation near the lower end of the muscular stomach and the rectum while preserving intestinal blood supply, as described previously [15]. The loops of the infection group were injected with 1 mL of the bacterial suspension containing 10^9^ CFU/mL, while the controls received LB broth only. The intestines were covered with a piece of gauze wetted with normal saline to retain humidity. The chickens were maintained under anesthesia for 2 h, 6 h, 8 h, and 12 h. Afterwards, they were euthanized using T61 intravenously and the intestines were removed for examination. Histological samples were fixed for 48 h in 10% neutral buffered formalin, embedded in paraffin, and processed using standard techniques for haematoxylin and eosin or immunohistochemistry staining. Rabbit anti-*S*. *flexneri* immunoglobulin G (IgG) (1∶200; Tianrun Biotech, China) was used as the primary antibody and goat anti-rabbit IgG labeled with horseradish peroxidase (1∶200; Bioss, China) was used as the secondary antibody in the immunohistochemical staining. The invasive capacity of the strain ZD02 within the intestinal epithelial cells was observed by TEM.

### Invasiveness of the *Shigella* strain ZD02 in primary chicken intestinal epithelial cells

Primary chicken intestinal epithelial cells were prepared and cultured as described previously [16]. The cells were cultured in 6-well plates for 48 h and washed three times with PBS. A 200-µL aliquot of the bacterial suspension diluted with DMEM/F12 at a density of 3×10^7^ CFU/mL was added to each cell and the plates were further incubated at 37°C under 5% CO_2_ for 1 h, 2 h, 3 h, and 4 h. The cells were then collected and examined to determine the invasive rate of the strain ZD02 in chicken intestinal epithelial cells using immunohistochemistry. Rabbit anti-*S. flexneri* IgG (1∶200, Tianrun Biotech) was used as the primary antibody and goat anti-rabbit IgG labeled with horseradish peroxidase (1∶200, Bioss) was used as the secondary antibody. Ten fields were selected and observed for each well under light microscopy. The invasive rate was calculated as the number of infected cells/total cells ×100%. Scanning electron microscopy (SEM; S-4800; Hitachi, Japan) [17] and TEM (JEM-1400) were used to examine the interactions between the invaded bacteria and epithelial cells.

## Results

### Virulence of the *Shigella* strain ZD02

**Figure 1:**
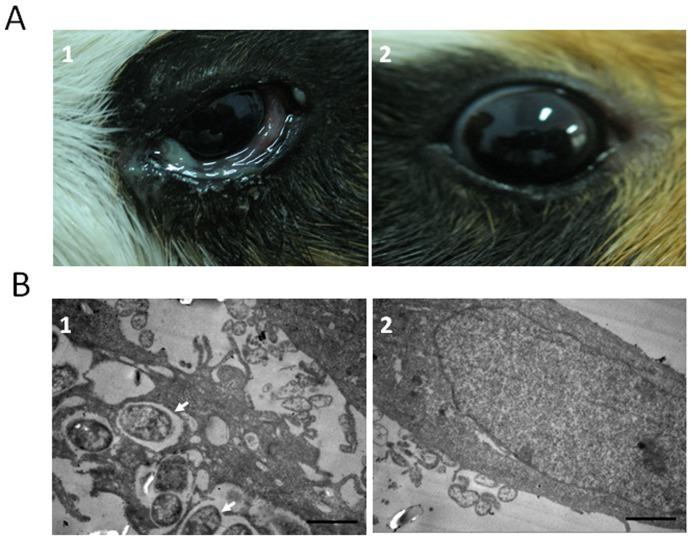
The virulence of the human *Shigella* strain ZD02 using the Sereny test and the HeLa cell invasiveness test. (A) Sereny test. Guinea pigs were infected with *Shigella* strain ZD02 via the cornea and conjunctiva. Corneal and conjunctiva inflammation were seen in infected eyes at 18 h post-inoculation (A1). Corneas and conjunctiva is normal in uninfected control eyes (A2). (B) HeLa cells infected with the *Shigella* strain ZD02. Transmission electron microscopy was used to detect the internalized bacteria within HeLa cells. Representative pictures are shown. The internalized bacteria (arrowheads) were detected at 2 h post-inoculation (B1). Control HeLa cells without infection (B2). Scale bars = 1 µm.

The infected guinea pigs developed edematous eyelids, congestive conjunctiva, cloudy corneas, and purulent discharge 18 h after infection with the *Shigella* strain ZD02 (Figure 1 A1). The clinical signs became exaggerated at 40 h. No significant changes were noticed in the control animals (Figure 1 A2). TEM showed that *Shigella* invaded the HeLa cells (Figure 1 B1). These results show that the human *Shigella* strain ZD02 is both invasive and virulent.

### Chicken infected with the *Shigella* strain ZD02

**Figure 2:**
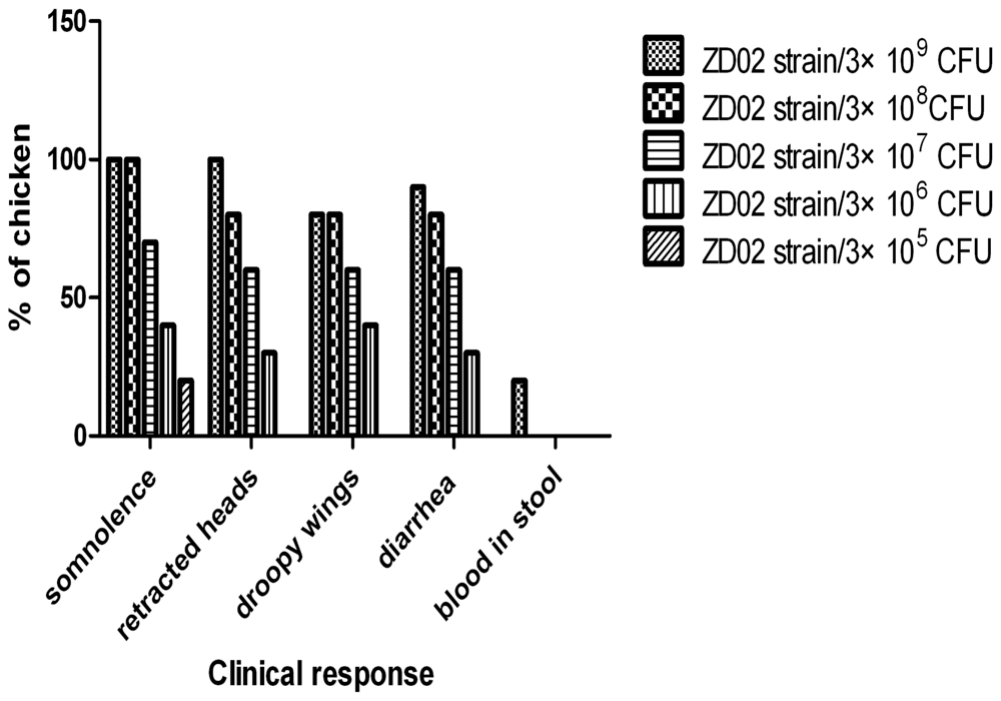
Clinical responses of chickens after intraperitoneal injection. Each bar denotes number of the clinical response/total in each experimental group after 12 h of intraperitoneal injection with *Shigella* strain ZD02.

In the intraperitoneal injection group, the chickens huddled together post-infection and demonstrated clinical signs including severe depression, weakness, somnolence, droopy wings, retracted heads, ruffled feathers, loss of appetite, and standing still with eyes closed at 6 h as well as diarrhea and pasting of vent feathers at 12 h (Figure S1 and Figure 2). Deaths occurred in subgroups I, II, and III at 48–96 h post-infection. There were not deaths in subgroups IV and V, or rather only depression and hypersomnia were observed. An additional file shows this in detail (Table S2). On postmortem examination, findings in chickens of the intraperitoneal injection group included: degeneration and yellow color of the liver; hemorrhage within the heart, lung, and spleen; intestinal edema; and fluid and gas accumulation within the cecum. The bacteria could be isolated from organs of dead chickens and stool or cloaca of infected chickens. The significant clinical signs and death weren't observed, and the bacteria could not be isolated from stool or cloaca in the crop injection groups. Clinical manifestations and dissection findings in the controls were normal. The LD_50_ of *Shigella* infection via intraperitoneal injection in the three-day-old chickens was 1.19×10^8^ CFU.

### Histopathology of SPF chickens infected with the *Shigella* strain ZD02 via ligated intestinal loop

**Figure 3:**
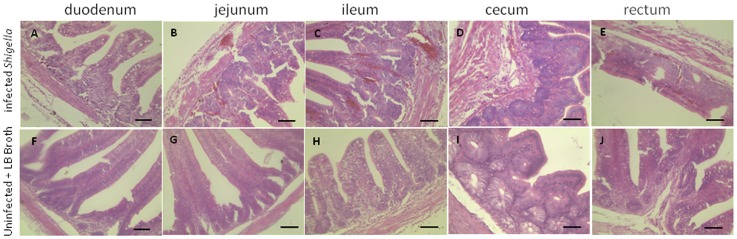
Histopathology of the intestine of one-day-old SPF chickens infected with the *Shigella* strain ZD02 via ligated intestinal loop. The duodenum, jejunum, ileum, and cecum from *Shigella*-infected chickens showed congestion, edema, and neutrophil infiltration (A–E, respectively). No significant pathological changes were noticed in the uninfected controls (F–J, respectively). The sections were stained with hematoxylin and eosin. Representative pictures are shown. Scale bars = 50 µm.

One-day-old SPF chickens were infected with *Shigella* strain ZD02 via ligated intestinal loop. Various degrees of intestinal hemorrhage and edema were noted in the infected chickens (Figure S2). At 12 h post-inoculation, severe congestion and edema were seen in the jejunum and ileum accompanied by neutrophil infiltration (Figure 3B, 3C). Neutrophil infiltration and mild congestion was seen in the duodenum as well (Figure 3A), while the cecum and rectum displayed only mild congestion (Figure 3D, 3E). Red blood cells, necrotic epithelial cells, cell debris, and protein-like materials were found in the intestinal cavity. No anomalies were found in the control chickens.

### Invasiveness of the *Shigella* strain ZD02 within the chicken intestine

**Figure 4:**
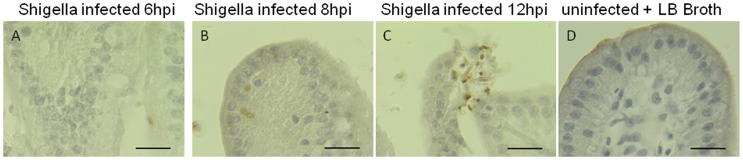
Immunohistochemistry of the jejunum of one-day-old SPF chickens infected with the *Shigella* strain ZD02 via ligated intestinal loop. Bacterial invasion (brown staining) was noticed in the epithelial cells 6 h (A) and 8 h (B) post-inoculation. The intestinal villus detached and the bacteria (brown staining) were released from the lysed epithelial cells (C). Intestinal villus of uninfected chicken was shown as control (D). Scale bars = 20 µm.

**Figure 5:**
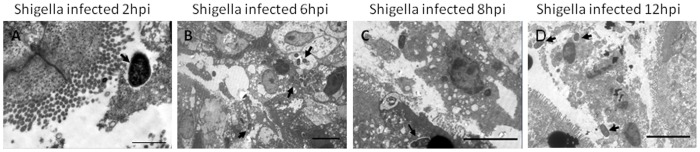
Transmission electron microscopy of the jejunum of one-day-old SPF chickens infected with the *Shigella* strain ZD02 via ligated intestinal loop. *Shigella* (arrowheads) were found near the epithelial cells at 2 h post-inoculation (A. Scale bar = 1 µm). Internalized bacteria (arrowheads) in the epithelial cells were found at 6 h (B. Scale bar = 5 µm) and 8 h (C. Scale bar = 5 µm). The epithelial cells started to lyse and release bacteria (arrowheads) at 12 h (D. Scale bar = 5 µm).

Immunohistochemistry studies of the chicken intestines infected with *Shigella* strain ZD02 via the ligated intestinal loop showed that the bacteria were not internalized within the epithelial cells of the jejunum and ileum at 2 h post-inoculation; nevertheless, it was noticed at 6–8 h (Figure 4A, 4B). Detached intestinal villi, necrotic epithelial cells, and released *Shigella* cells were noted at 12 h (Figure 4C). No internalized bacterial cells were noticed in the epithelial cells of the duodenum, cecum, or rectum. Electron microscopy showed that *Shigella* attached to the surfaces of the intestinal epithelial cells 2 h post-inoculation (Figure 5A), while internalized bacteria were found at 6 and 8 h (Figure 5B, 5C). Intestinal epithelial cells had lysed at 12 h and released the bacteria (Figure 5D). Only epithelial cells in the jejunum and ileum had internalized bacteria.

### Invasiveness of the *Shigella* strain ZD02 in primary chicken intestinal epithelial cells

**Figure 6:**
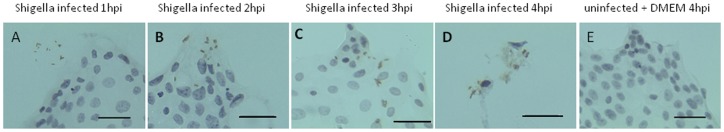
Immunohistochemistry of the primary chicken intestinal epithelial cells infected with the *Shigella* strain ZD02. Uninternalized bacteria (brown staining) were seen near the epithelial cells at 1 h post-inoculation (A). Internalized bacteria (brown staining) were found at 2 h (B) and 3 h (C). The lysed epithelial cells released bacteria (brown staining) at 4 h (D). Uninfected epithelial cells were shown as control (E). Scale bars = 20 µm.

**Figure 7:**
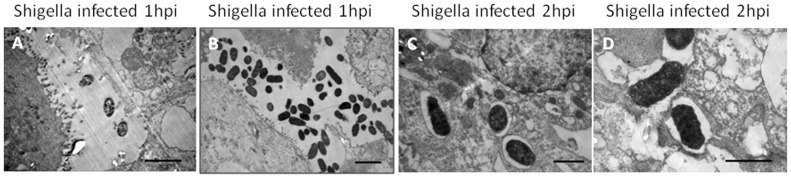
Transmission electron microscopy of the primary chicken intestinal epithelial cells infected with the *Shigella* strain ZD02. Uninternalized bacteria were seen near the epithelial cells at 1-inoculation (A, B. Scale bars = 2 µm). The internalized bacteria were encapsulated with phagosome-like membranes (C, D. Scale bars = 1 µm).

**Figure 8:**
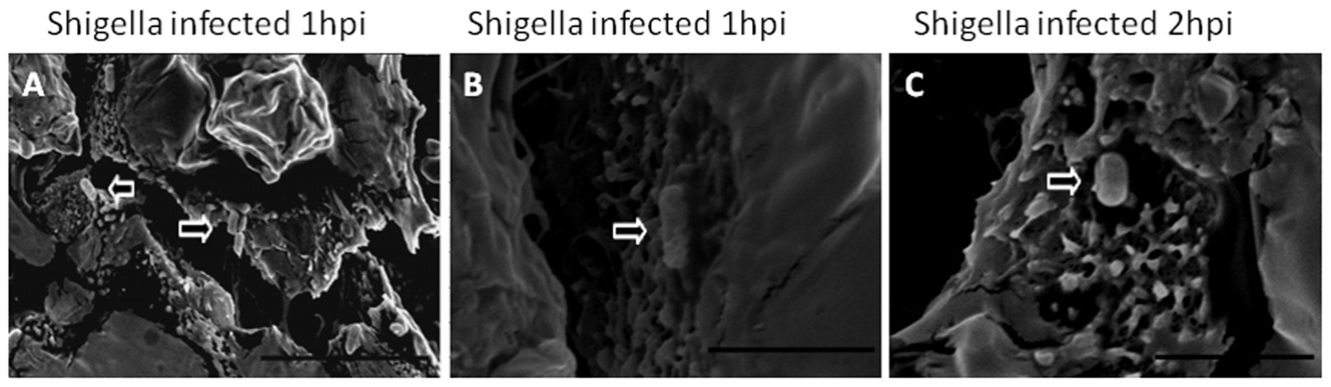
Scanning electron microscopy of the primary chicken intestinal epithelial cells infected with the *Shigella* strain ZD02. Uninternalized bacteria (arrowhead) were seen near the epithelial cells 1 h post-inoculation (A. Scale bar = 10 µm). Bacteria (arrowheads) attached to the surfaces of the epithelial cells at 1 h (B. Scale bar = 3 µm). Bacteria (arrowheads) were being internalized by the epithelial cells at 2 h (C. Scale bar = 3 µm).

Primary chicken intestinal epithelial cells infected with the *Shigella* strain ZD02 were examined using immunohistochemistry, TEM, and SEM. The immunohistochemistry study showed that *Shigella* could invade the chicken epithelial cells as early as 2–3 h post-infection (Figure 6B, 6C) and the infected cells started to lyse and release the bacteria at 4 h (Figure 6D). The number of invading *Shigella* increased with infection time and the invasive rate reached up to 11.2±0.43% at 4 h. TEM showed that internalized bacteria within the chicken epithelial cells at 2 h were encapsulated by phagosome-like membranes (Figure 7C, 7D). SEM showed that the bacteria attached to the epithelial cell surfaces at 1 h (Figure 8A, 8B) and started becoming internalized the epithelial cells at 2 h (Figure 8C).

## Discussion

*Shigella* is a pathogen with constrained hosts. However, its host range has showed to expand in recent years. The pathogenesis of *Shigella* is attributed to the organism's ability to invade, replicate and spread intercellularly within the colonic epithelium. Virulent *Shigella* can invade intestinal epithelial cells, causing dysentery and other intestinal clinical signs in hosts [18]. *Shigella* virulence can be usually assessed using the Sereny test [12], HeLa cell invasiveness test [14], and Congo red binding assay [19]. This study has demonstrated the virulence of the human *Shigella* strain ZD02 using the Sereny test and the HeLa cell invasiveness test.

Given that *Shigella* is tissue specific organism which always invade large intestine, we have ever infected chicken with *Shigella* strain ZD02 via the intrarectal route. Immunohistochemistry and electron microscopy showed that there wasn't any internalized bacterium in large intestine. The inoculation of *Shigella* in SPF chickens via intraperitoneal injection caused severe clinical signs and even death, suggesting that *Shigella* is pathogenic in chickens. In contrast, the inoculation of *Shigella* via crop injection caused depression merely. We speculate that *Shigella* can't reach the intestines of chickens with normal immune function via crop injection “safely”, therefore, no significant clinical signs were induced in this group. However, the specific mechanisms require further study.

*Shigella* can invade human epithelial cells, proliferate, and then spread, which is the critical process of its pathogenicity and the mechanism for its virulence [18]. Complex interactions are involved in the invasion of *Shigella* into the intestinal barrier, such as activating some signaling pathways to interrupt target cells, which plays an important role in its pathogenesis [20]–[22]. Therefore, the SPF chicken intestine and primary chicken epithelial cells were infected with *Shigella* strain ZD02 to investigate its invasiveness.

Multiple animal models of shigellosis have been established. Rabbani et al [23] infected the rabbit intestine *with Shigella* using the ligated ileal loop assay and found that it caused inflammation within the colon. Fernandez et al [24] established a mouse model of shigellosis by orally infecting four-day-old mice with *Shigella* and found similar pathological changes to those of human bacterial dysentery and inflammation. Martino et al [25] provided a streptomycin-treated murine model in which *Shigella* are able to reach their natural tissue target: colon. Shim et al [26] established a guinea pig model by inoculating *S. flexneri* 2α or 5α in the rectum. Jeong et al [27] established a piglet model of acute gastroenteritis with *Shigella* type I and found that piglets are highly sensitive to *Shigella* and demonstrate clinical signs such as acute diarrhea, anorexia, and dehydration. Barman et al [28] established a shigellosis in the guinea-pig model infected with *Shigella dysenteriae* into the cecocolic junction after ligation of the distal cecum without any preparatory treatment, which induced acute inflammation. Yang et al [29] established an adult mice model of Shigellosis by intraperitoneal infection. This study was the first to demonstrate that *Shigella* strain ZD02 can invade epithelial cells in the jejunum and ileum of one-day-old SPF chickens via ligated intestinal loop inoculation and cause severe congestion and edema. Therefore, *Shigella* can cause intestinal damage and disease after it invades the jejunum and ileum of chickens in some cases.

To further investigate the invasiveness of *Shigella* isolated from human in chickens, we infected isolated primary chicken epithelial cells with the strain ZD02. These cells became contaminated with fibroblastic cells and could not reach 100% confluence in 48-well plates. In addition, infected cell morphology was inconsistent among different wells. Therefore, the classical Gentamicin protection assay could not be used to determine the invasion rate of *Shigella* isolated from human within chicken intestinal epithelial cells. Instead, we used immunohistochemistry to determine the invasiveness of the strain ZD02 into cells and calculated the invasion rates. The results showed that *Shigella* isolated from human can invade the chicken intestinal epithelial cells. The invasion rate increased with infection time and reached 11.2±0.43% at 4 h post-inoculation, confirming the invasive capacity of *Shigella* in chicken intestinal epithelial cells.

*Shigella* is transmitted primarily via the fecal-oral route. This study showed that *Shigella* is pathogenic in chickens and invasive in chicken intestinal epithelial cells. However, *Shigella* could induce depression but didn't cause intestinal clinical signs such as diarrhea and blood dysentery via crop injection. We found that *Shigella* was less pathogenic in chickens via natural infection route than that in human because *Shigella* has difficulty in invading the chicken intestinal barrier and escaping immune clearance. *Shigella* can circumvent a host's innate immune system via versatile pathogenic mechanisms [18]. Similarly, adult mice are resistant to oral *Shigella* infection. However, intraperitoneal challenge with virulent *S. flexneri* 2a resulted in diarrhea and severe body weight loss in adult B6 mice. The bacteria could invade and colonize not only systemic tissues but also the serosa and lamina propria region of the large intestine [29]. *Shigella* in the peritoneal cavity which was injected into SPF chickens via intraperitoneal route attached and colonized in the serosa and muscle layer of the chicken intestine and entered the lamina propria region successfully, then caused death and other clinical symptoms. So, the intraperitoneal route may be also a promising entry site for *Shigella* which results in shigellosis–like symptoms in SPF chicken. Endogenous flora could protect the intestinal tissue from *Shigella* infection, so adult mice are resistant to oral *Shigella* infection. However, treatment of adult mice with streptomycin is a prerequisite for colonization of the intestinal tract by *Shigella*. The colon and eventually the cecum of the streptomycin-treated adult mice were the main targets for the invasion of *Shigella* [25]. Antibiotic usage is known to be rampant in the poultry industry which may cause enteric dysbacteriosis. We speculated that *Shigella* can cause disease if it invades the chicken intestines in the presence of immune suppression or enteric dysbacteriosis. Such cases usually happen in poultry production. In fact, this might be the reason for the occurrence of clinical chicken shigellosis in China.

Microbes have adapted many fascinating strategies to co-evolve with their hosts. There is a constant molecular interplay between host factors and invading pathogens. The microbes can obtain a range of host factors by horizontal gene transfer or rearrangement [30], [31] as well as loss or deactivation of unimportant or even deleterious genes [32] to escape immune clearance, or even adapt [33] by switch [30] or jump [34], [35] within a new host. We have ever tried to identify the origin of *Shigella* isolated from chicken by comparing the sequences of eight housekeeping genes (*thrB/thrC*, *trpC/trpB*, *purM/purN*, *mdh/argR*) between *Shigella* isolated from human and chicken previously. The results showed that *Shigella* isolated from human or chicken is highly homogeneous without uncertain evolutionary evidence [10].

We have previously detected the expression of receptors CD44 and α5β1 in chicken intestinal epithelial cells, which can serve as the basis for the invasion of intestinal epithelial cells and *Shigella* pathogenesis [36], [37], [38]. A variety of factors, such as human intervention, the stress-inducing breeding environment, co-infection of pathogens, and immune dysfunction, may confer the *Shigella* with invasiveness and pathogenicity within chickens without the apparent need for host-adapting evolution, resulting in human–poultry cross-infection.

In conclusion, *Shigella* can invade chicken intestinal epithelial cells in vitro and chicken intestinal mucosa in vivo. Meanwhile, receptors required for the entry of *Shigella* are detected in chicken intestinal epithelial cells [38]. Consequently, it is possible for *Shigella* to infect or even kill chickens under certain condition. The findings suggest that *Shigella* isolated from human or chicken have highly similar pathogenicity as well as the possibility of human–poultry cross-infection, which is of public health significance.

## Supporting Information

Figure S1 **The symptoms of SPF chickens infected with the** ***Shigella*** **strain ZD02 via intraperitoneal injection.** The chickens showed depression (A), dysentery and pasting vent (B).
(DOC)

Figure S2 **Gross pathology of the SPF chicken intestines infected with the** ***Shigella*** **strain ZD02 at 12 h post-inoculation.** The intestine of chicken infected with *Shigella* ZD02 showed severe congestion and edema (upper panel). No significant pathological changes were noted in the uninfected controls (lower panel).
(DOC)

Table S1 **Detailed information of chicken infected with** ***Shigella*** **strain ZD02 via intraperitoneal or crop injection.**
(DOC)

Table S2 **Detailed incidence and mortality of three-day-old SPF chickens infected with the** ***Shigella*** **strain ZD02 via intraperitoneal injection.** The LD_50_ of *Shigella* infection via intraperitoneal injection in the three-day-old chickens was 1.19×10^8^ CFU using the Reed-Muench method.
(DOC)
