# Supplementary material for: Pathogenicity of Shigella in Chickens
Source: PLoS One. 2014 Jun 20;9(6):e100264. doi: 10.1371/journal.pone.0100264 (PMC4064985; doi:10.1371/journal.pone.0100264)
Supplement: Figure S1 — The symptoms of SPF chickens infected with the Shigella strain ZD02 via intraperitoneal injection. The chickens showed depression (A), dysentery and pasting vent (B). (DOC) [file pone.0100264.s001.doc]

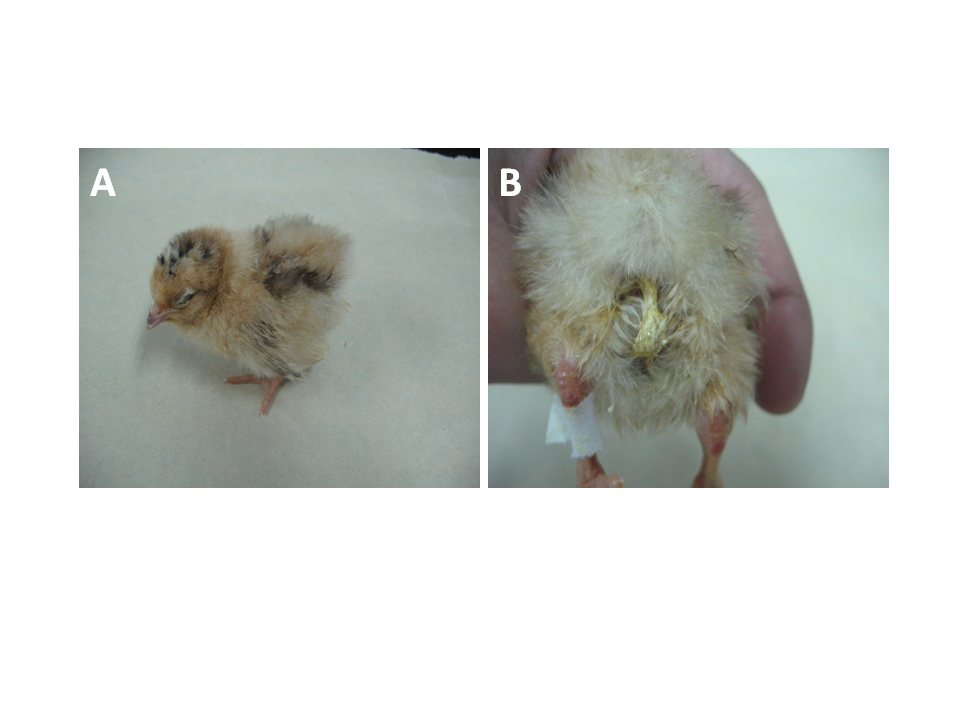
 Figure S1. **The symptoms of specific pathogen-free chickens infected with the *Shigella* ZD02 strain via intraperitoneal injection**. The chickens showed depression (A), dysentery and pasting vent (B).
