# Supplementary material for: Pathogenicity of Shigella in Chickens
Source: PLoS One. 2014 Jun 20;9(6):e100264. doi: 10.1371/journal.pone.0100264 (PMC4064985; doi:10.1371/journal.pone.0100264)

Figure S2. **Gross pathology of the specific pathogen-free chicken intestines infected with the *Shigella* ZD02 strain at 12 h post-inoculation.** The intestine of chicken infected with *Shigella* ZD02 showed severe congestion and edema (upper panel). No significant pathological changes were noted in the uninfected controls (lower panel).


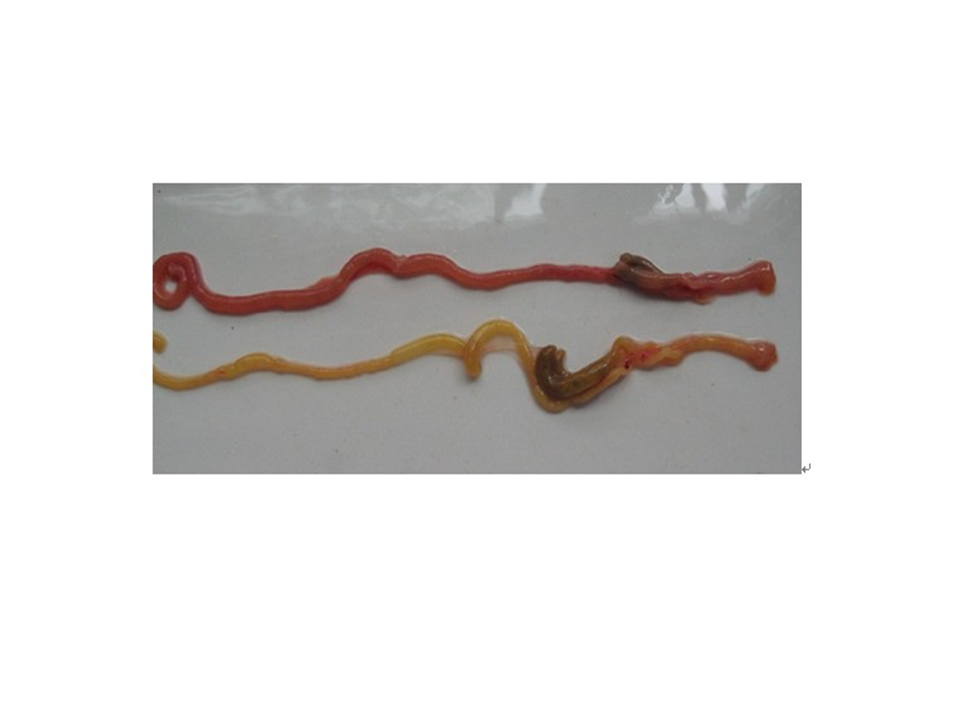

Supplement: Figure S2 — Gross pathology of the SPF chicken intestines infected with the Shigella strain ZD02 at 12 h post-inoculation. The intestine of chicken infected with Shigella ZD02 showed severe congestion and edema (upper panel). No significant pathological changes were noted in the uninfected controls (lower panel). (DOC) [file pone.0100264.s002.doc]
