# Supplementary material for: Pathogenicity of Shigella in Chickens
Source: PLoS One. 2014 Jun 20;9(6):e100264. doi: 10.1371/journal.pone.0100264 (PMC4064985; doi:10.1371/journal.pone.0100264)
Supplement: Table S1 — Detailed information of chicken infected with Shigella strain ZD02 via intraperitoneal or crop injection. (DOC) [file pone.0100264.s003.doc]

Table S1: Detailed information of chicken infected with *Shigella* ZD02 strain via intraperitoneal or crop injection.

| Intraperitoneal injection groups | Inoculation dosage  (CFU) | Inoculated chickens  (n) | Crop injection groups | Inoculation dosage  (CFU) | Inoculated chickens  (n) |
| --- | --- | --- | --- | --- | --- |
| I | 3×10^9^ | 10 | Ⅵ | 3×10^9^ | 10 |
| II | 3×10^8^ | 10 | Ⅶ | 3×10^8^ | 10 |
| III | 3×10^7^ | 10 | Ⅷ | 3×10^7^ | 10 |
| IV | 3×10^6^ | 10 | Ⅸ | 3×10^6^ | 10 |
| V | 3×10^5^ | 10 | Ⅹ | 3×10^5^ | 10 |
| Control | LB broth | 5 | Control | LB broth | 5 |
