# Supplementary material for: Pathogenicity of Shigella in Chickens
Source: PLoS One. 2014 Jun 20;9(6):e100264. doi: 10.1371/journal.pone.0100264 (PMC4064985; doi:10.1371/journal.pone.0100264)
Supplement: Table S2 — Detailed incidence and mortality of three-day-old SPF chickens infected with the Shigella strain ZD02 via intraperitoneal injection. The LD50 of Shigella infection via intraperitoneal injection in the three-day-old chickens was 1.19×10^8^ CFU using the Reed-Muench method. (DOC) [file pone.0100264.s004.doc]

Table S2. Detailed incidence and mortality of three-day-old specific pathogen-free chickens infected with the *Shigella* ZD02 strain via intraperitoneal injection

| Groups | Inoculation dosage  (CFU) | Inoculated chickens  (n) | Results | | Cumulative Results | | | |
| --- | --- | --- | --- | --- | --- | --- | --- | --- |
|  |  |  | Death  (n) | Survival (n) | Death  (n) | Survival (n) | Total (n) | Mortality (%) |
| I | 3×10^9^ | 10 | 10 | 0 | 19 | 0 | 19 | 100.0 |
| II | 3×10^8^ | 10 | 6 | 4 | 9 | 4 | 13 | 69.2 |
| III | 3×10^7^ | 10 | 3 | 7 | 3 | 11 | 14 | 21.4 |
| IV | 3×10^6^ | 10 | 0 | 10 | 0 | 21 | 21 | 0 |
| V | 3×10^5^ | 10 | 0 | 10 | 0 | 31 | 31 | 0 |
| Control | LB broth | 5 | 0 | 5 | 0 | 5 | 5 | 0 |
